# Assessment of diabetes self-management amongst Nigerians using the diabetes self-management questionnaire: a cross-sectional study

**DOI:** 10.11604/pamj.2021.40.178.28584

**Published:** 2021-11-24

**Authors:** Ogochukwu Chinedum Okoye, Oluwatoyin Abisoye Ohenhen

**Affiliations:** 1 Department of Internal Medicine, Delta State University, Abraka, Delta State, Nigeria,; 2 Department of Internal Medicine, University of Benin Teaching Hospital, Benin City, Nigeria

**Keywords:** Diabetes mellitus, self-management, diabetes self-management questionnaire, glycaemic control, glycated haemoglobin

## Abstract

**Introduction:**

self-management is probably the most important factor contributing to achieving euglycaemia. The Diabetes Self-Management Questionnaire (DSMQ) is an instrument that shows favourable prospects compared to older measures. This study aimed to investigate the association between self-management and glycaemic control using the DSMQ, and determine factors that affect glycaemic control in patients living with diabetes mellitus.

**Methods:**

a cross-sectional analytic study of 103 patients, carried out in a public tertiary health institution located in a Southern Nigerian City. An interviewer administered DSMQ was used to assess self-management among the patients. Data analysis was performed using SPSS 22.0.0, and AMOS 22.0.0 (IBM SPSS Statistics, New York, USA).

**Results:**

females had significantly lower DSMQ scores compared to males (40 vs. 36, P=0.015) while median DSMQ score was highest in participants with tertiary level of education (P=0.017), and those who earned the highest annual income (P=0.007). The DSMQ´s behaviour scales showed a notable negative correlation with HbA1c (-0.565, P < 0.001). More females (80.3%) than males (56.3%) had high HbA1C (X^2^=6.44, P=0.016).

**Conclusion:**

diabetes self-management using DSMQ showed significant correlation with glycaemic control. Male sex, higher income, and higher level of education are associated with better self-management and glycaemic control.

## Introduction

Self-management is probably the most important factor contributing to achieving euglycaemia [1-3] and several self-report measures have been developed in the past for the assessment of self-care among patients with diabetes mellitus [4-6]. The Diabetes Self-Management Questionnaire (DSMQ) is a relatively new tool used to assess diabetes self-management. The scale covers several important domains including diet, medication adherence, blood glucose monitoring, physical activity and contact with health-care professionals which are activities related to glycaemic control. The DSMQ is a reliable and valid instrument that enables an efficient assessment of self-care behaviours associated with glycaemic control and shows favourable prospects compared to older measures [7-9]. Although this instrument has been thoroughly evaluated and used in Europe [10-14], to our knowledge, no local study has investigated the use of DSMQ on Nigerians. This study aimed to investigate the association between self-management and glycaemic control using the DSMQ, and determine factors that affect glycaemic control in patients living with diabetes mellitus. We hypothesize that more desirable diabetes self-management is associated with good glycaemic control, and that socio-demographic factors such as poor level of education and low income will negatively affect glycaemic control.

## Methods

### Study design and patients

The study was of cross-sectional analytic design, carried out in a public tertiary hospital located in a major city in Southern Nigeria. The hospital has a robust endocrinology unit with four endocrinologists who run outpatient clinics two days a week and about 40 patients with diabetes mellitus are seen weekly. Patients living with type 1 or 2 diabetes mellitus were eligible for the study, however only those willing and able to give consent were recruited; patients who were too ill to respond to questions were excluded. The calculated sample size using the Cochran formula was 168 (a 5% margin of error, 95% confidence level, a response distribution of 25% and the known population of diabetics in the study centre, which is 400). Assuming a non-response rate of 10%, 184 patients were considered as adequate sample. Patients were recruited through the outpatient clinic over a period of 13 weeks. Sample selection was by the systematic sampling technique using a sampling interval of five. Case definition for diabetes mellitus was based on clinical diagnosis recorded in the participants´ case files; both type 1 and 2 diabetics were included.

The patients´ socio-demographic data, data on health status, duration of diagnosis, current management of diabetes, and presence of complications were collated using a data collection sheet; this information was obtained from the patients´ self-report as well as the patients´ case files to minimise information bias. The DSMQ was used to assess self-management among the patients, and it was interviewer-administered for participants that were illiterate and self-administered for participants that were literate. HbA1c was measured for all patients using the Biorad D10 HbA1c Automatic analyzer. A HbA1c ≥ 7.0% was regarded as poor glycaemic control. The Diabetes Self-Management Questionnaire (DSMQ) is an instrument used in analysing self-reported behavioural problems related to poor glycaemic control. There is evidence of above-average convergence between the self-management behaviours measured by the DSMQ and glycaemic control, which suggests good eligibility and utility of this tool either for analysing behavioural problems related to hyperglycaemia in clinical practice or for testing putative mediation of the effect of a given factor on glycaemic control by diabetes self-management [8].

The DSMQ consists of 16 items spanning different domains of diabetes self-management; these items describe different behaviours related to diabetes self-care, reflecting five main areas- patients’ dietary control, medication adherence, blood glucose monitoring, physical activity and physician contact [7]. Referring to the previous eight weeks, participants rate the extent to which each description applies to them on a four-point Likert scale, where 0= ‘does not apply to me’, 1= ‘applies to me to some degree’, 2= ‘applies to me to a considerable degree’, and 3= ‘applies to me very much’. ‘Item scores are transformed so that higher scores indicate more desirable self-management behaviour (requiring reverse-scoring of negatively-keyed items) and summed/transformed to five scale scores with ranges from 0 to 10’ [7].

### Ethical considerations

Ethical approval for this study was obtained from the hospital Health Research and Ethical Committee (HREC). All patients were informed about the study aims, procedures and risks and signed an informed consent prior to inclusion. Codes were used as participant identifiers to ensure privacy and confidentiality.

### Data analysis

Data analysis was performed using SPSS 22.0.0, and AMOS 22.0.0 (IBM SPSS Statistics, New York, USA). Only adequately completed questionnaires were included in the analysis. The Mann-Whitney U test, and the Kruskal-Wallis H test were used to examine the association between median DSMQ scores and socio-demographic and health factors. A logistic regression model was performed to determine the independent risk factors for poor DSMQ scores; variables considered included sex, level of education, annual income, and type of diabetes treatment. The DSMQ total scores were divided according to the median into two groups. Participants who scored less than or equal to the median were categorized as having “poor” DSMQ scores and assigned a score of “1” while those who scored more than the median were categorised as having “good” DSMQ scores and scored “0” [15]. P < 0.05 and 95% CI were considered statistically significant.

The main statistical analysis was determining the association between self-management behaviour and HbA1C. Structural equation modelling was performed using maximum likelihood estimation. The basic test model included the variables HbA1c and diabetes self-management, with the latter being modelled as a latent variable operationalised by the DSMQ´s five self-management behaviours. This approach enabled the integration of the heterogeneous behaviours in a single variable of diabetes self-management, while the individual behaviours were weighted based on the fit to the data. The structural equation modelling therefore warranted optimal predictive power of diabetes self-management, as operationalised by the DSMQ behaviours regarding glycaemic control.

The resulting models were tested on all patients (type 1 and type 2 diabetics), and revised by successively modelling correlations between the variables´ error terms, following significant modification indices (threshold = 4.0) in order to attain adequate fit of the models. Model fit was evaluated according to the recommendations by Hu and Bentler [16]; Standardised Root Mean Square Residual (SRMR) =0.08, Comparative Fit Index (CFI) =0.95 and Root Mean Square Error of Approximation (RMSEA) =0.06 (with upper bound of the 90% CI 0.08). The fitted models were then used to evaluate the predictive power of the DSMQ regarding HbA1c (assessed by the explanation of variance, i.e. squared multiple correlation, of HbA1c).

## Results

One hundred and three patients with adequately completed questionnaires were studied (response rate=61.3%), 68.9% were females. Mean age was 59.9± 12.8years. Only five out of 103 patients had Type 1 diabetes mellitus. Overall median duration of diagnosis was 10 years (IQR=3) years. Median duration of treatment was 7 years (1QR=2). Socio demographic and health status data of the participants are shown in Table 1 and Table 2. Mean HbA1c for all participants was 8.9 ± 2.3%, 72.8% of participants had HbA1c ≥ 7.0%. Significantly more females (80.3%) than males (56.3%) had high HbA1C (X^2^=6.44, P=0.016). Level of education was associated with HbA1C, 88% of those with primary level of education compared to 79.1% and 55.9% of those with secondary and tertiary level of education respectively had high HbA1c level (X^2^=11.37, P=0.006). There was no significant correlation between age and HbA1C (R=0.008, P=0.934). Income was not associated with HbA1C levels (X^2^=0.760, P=0.872).

**Table 1:** sociodemographic characteristics of 103 participants

Demographic Variable	n (%)
**Age (Years)**	
20-29	1 (1.0)
30-39	8 (7.8)
40-49	11 (10.7)
50-59	27 (26.2)
60-69	33 (32.0)
70-79	20 (19.4)
80-89	2 (1.9)
90-99	
**Sex**	1 (1.0)
Male	32 (31.1)
Female	
**Level of Education**	71 (68.9)
Primary	25 (24.3)
Secondary	43 (41.7)
Tertiary	34 (33.0)
None	
**Annual Income ($)**	1 (1.0)
<$2000	40 (35.9)
$2000- <$4000	22 (17.5)
$4000- <$8000	35 (34.0)
>$8000	6 (5.8)
Does not want to disclose	9 (6.8)

**Table 2:** DSMQ scores according to socio-demographic and clinical parameters of the patients

Variable	F(n=103)	Median (IQR)*	P
**Sex**			0.015
Male	32	40.0 (34.3-43.0)	
Female	71	36.0 (31.0-40.0)	
Age(yrs)			0.944
20-39	9	38.0 (31.0-41.0)	
40-69	38	37.0 (33.0-41.5)	
≥70	56	37.0(33.3-40.8)	
**Level of education**			0.017
Primary	26	35.0(28.3-39.3)	
Secondary	43	37.0(33.0-40.0)	
Tertiary	34	39.0(35.0-43.0)	
**Annual Income**			0.007
<$2000	40	36.5(31.0-40.0)	
$2000- <$4000	22	34.0(30.0-39.3)	
$4000- <$8000	35	35.0(35.0-43.0)	
>$8000	6	37.5(32.3-43.5)	
Type of Diabetes			0.406
Type I	5	33.0(31.0-40.0)	
Type II	98	37.0(33.0-41.0)	
**Type of Treatment**			
Exclusive Insulin	3	37.0(31.0- )	0.250
Combined with oral medication	20	39.5(35.0-43.0)	
Oral Medication	80	36.5(31.3-40.8)	
Hypertension			
Yes	28	35.5(30.8-40.0)	0.363
No	75	38.0(33.0-41.3)	
**Duration of Diabetes**			0.524
<5 years	30	38.0(33.7-41.5)	
5-10 yrs	31	38.0(35.0-41.0)	
>10 years	42	35.5(31-40.5)	
Number of Complications*			0.489
0	29	40.0(32.0-42.0)	
1	51	37.0(34.0-40.0)	
2	22	35.0(31.0-40.5)	
3	1	35.0(NR)	

* Complications included peripheral neuropathy, DM retinopathy, peripheral artery disease, DM nephropathy, glaucoma and cataract. NR=not required

The Median DSMQ score for all participants was 37.0 (33.0-41.0). Females had significantly lower DSMQ scores compared to males (40 vs. 36, P=0.015) while median DSMQ score was highest in participant with tertiary level of education (P=0.017), and those who earned the highest annual income (P=0.007) (Table 2). Females had 2.28 times the odds of having poor DSMQ scores compared to males, although this was not statistically significant at P=0.06. Those with primary education had 4 times the odds of having poor DSMQ compared to those with tertiary education (p=0.011). Those with an annual income of $4000- $8000 had 73% lesser odds of having poor DSMQ compared to those earning an annual income of $2000- $4000, this remained statistically significant after adjusting for confounding variables (Table 3).

**Table 3:** risk factors of poor DSMQ scores

	Crude OR for poor DSMQ			Adjusted OR for poor DSMQ		
	OR	95% CI	P	OR	95% CI	P
**Sex**						
Male	1.00	Reference		1.00	Reference	
Female	2.28	0.96 -5.36	0.06	2.96	0.79-5.11	0.140
**Level of Education**						
Primary	4.12	1.38-12.27	0.011	3.549	1.08-11.64	0.370
Secondary	2.10	0.83-5.31	0.114	1.869	0.69-5.03	0.216
Tertiary	1.00	Reference		1.000	Reference	
**Level of Income**						
<$2000	0.57	0.19-1.70	0.314	0.461	0.14-1.51	0.201
$2000- <$4000	1.00	Reference		1.000	Reference	
$4000- <$8000	0.27	0.08-0.85	0.025	0.296	0.09-0.94	0.040
$8000- <$20,000	0.40	0.07-2.92	0.416	0.775	0.10-5.62	0.801
**†Type of Treatment**						
Exclusive Insulin	1.00	Reference				
Combined with oral medication	0.26	0.02-3.51	0.317			
Oral Medication	0.61	0.05-7.01	0.692			

**Figure 1:**
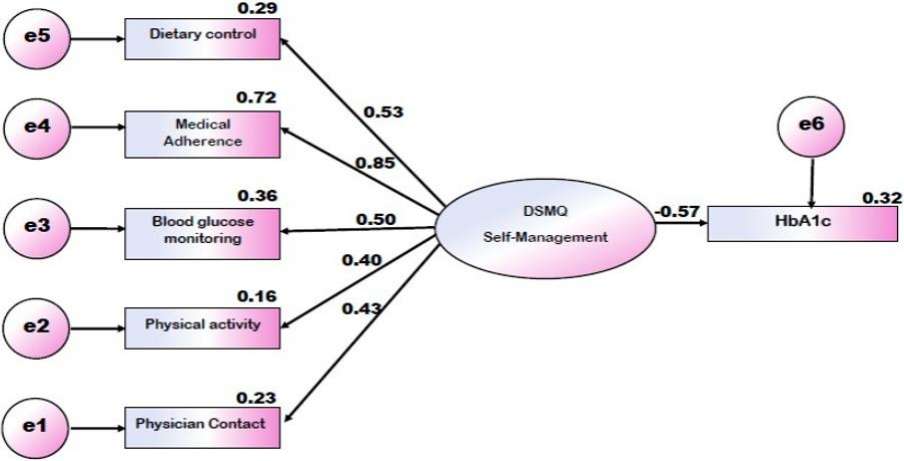
structural equation model of diabetes self-management as measured by the DSMQ

The structural equation model (SEM) of diabetes self-management as measured by the DSMQ is displayed in Figure 1 (SRMR=0.0646, CFI=0.921, RMSEA=0.10, IFI=0.925, GFI= 0.946, PNFI=0.52. Data are standardised regression weights (β) for paths or squared multiple correlations (R2) for variables. Boxes indicate manifest measurement variables; ovals indicate latent variables operationalised by manifest indicators; error terms are not displayed for ease of presentation. The model showed a good fit for the patients (Figure 1), and all regression weights were significant with P < 0.001. Diabetes self-management as operationalized by the DSMQ´s behaviour scales showed a notable negative association with HbA1c amounting to -0.565 (P < 0.001). The squared multiple correlations for these associations were 0.32 i.e. an explanation of 32% of glycaemic variation for the patients. Most relevant DSMQ behaviours with regard to glycaemic control were ‘medication adherence’ (β=0.85, p < 0.001), ‘blood glucose monitoring’ (β=0.60, p < 0.001), and ‘dietary control’ (β = 0.53, p < 0.001), whereas ‘physician contact’ (β=0.48, p < 0.001) and ‘physical activity’ (β=0.40, p < 0.001) appeared less relevant in this regard.

A HOELTER index of 93 (optimum: 200-75) indicates the adequacy of the sample size for this study. The Standardised Root Mean Square Residual (SRMR) was 0.06 (Adequate Fit), CMIN/DF=2.08 (Acceptable); Comparative Fit Index (CFI)=0.921 (Acceptable), indicating that 92% of the covariation in the data can be reproduced by the given model; IFI=0.925 (Acceptable); GFI= 0.946 (Good fit), Root Mean Square Error of Approximation (RMSEA) was 0.10, (poor fit), PNFI=0.52 (good fit); Overall the model could be regarded as a good fit.

## Discussion

The main objective of this study was to ascertain the association between self-management and glycaemic control amongst Nigerian diabetics using the Diabetes Self-Management Questionnaire. The DSMQ assesses five essential domains of diabetes self-management viz; dietary control, medication adherence, blood glucose monitoring, physical inactivity and physician contact. Amongst Nigerian diabetics, this study showed that the DSMQ has good psychometric. The Diabetes self-management of participants in this study showed a notable negative association with the glycated haemoglobin indicating that self-management of diabetes amongst study participants resulted in significantly lower HbA1c. This finding is in agreement with most reported research findings that persons with DM who practice self-care have good glycaemic control [6-8]. Furthermore, in assessing individual domain of DSMQ behaviour scales, medication adherence, blood glucose monitoring and dietary control all showed high correlation with HbA1c whereas, physical activity and physician contact were less relevant.

With regard to factors that affect glycaemic control, over two-thirds of the study participants had poor glycaemic control and this finding was similar to studies from South Western Nigeria [17] but higher than other studies from other regions in Nigeria [18,19]. This observed difference may be due to variations in study designs, and characteristics of the study populations. For instance, the mean duration of diabetes in the earlier study from South- South Nigeria was 8.5 ± 3.2 years while the median duration of DM in this present study is 10 years. Diabetes control worsens with increasing duration of diabetes due to many factors, including progressively declining beta islet cells.

The proportion of women with good glycaemic control was significantly lower than males using the International Diabetes Federation definition [20] of glycated haemoglobin less than 7%. Females also had significantly lower DSMQ scores and were 2.28 times more likely to have poorer DSMQ scores compared to males, signifying poorer diabetes self-management. The finding of poorer glycaemic control for women in this study is similar to findings in most studies which have reported significantly higher HbA1c levels in women and significantly fewer women than men achieve target HbA1c levels of <7 and <8% [21,22]. This observed difference between the females and males with regard to glycaemic control has been attributed to differences in regulation of glucose homeostasis (e.g, hormones and visceral adipose distribution) [22,23]. Other possible reasons for the different outcomes between men and women with respect to glycaemic control are treatment response (e.g, side effects) and psychological factors (eg, acceptance of disease) [24]. Salcedo-Rocha *et al*. in Mexico suggested that women had several social and economic disadvantages (i.e, lower education, lower participation in paid work, and reduced wages or economic dependence) that might decrease their ability to achieve glycaemic control successfully [25]. Other studies, however, have found no significant relationship between sex and glycaemic control. For example, a study of 180 patients with T2D from two health clinics in Texas in 2007 found no sex differences in glycaemic control, after adjusting for self-management behaviours and quality of life indicators, suggesting that sex differences in glycaemic control outcomes might be related to less perceived social support, less acceptance of disease and more difficulty in self-management behaviour in women [26].

Also, we noted that those with higher levels of education had significantly better glycaemic control, such that those with tertiary level of education had the highest median DSMQ scores and a similar trend was noted with the level of income, but the difference was not statistically significant. Similar findings with respect to level of education and glycaemic control have been reported by researchers in Saudi Arabia [27] and Netherlands [28] while in contrast, other studies reported that patients with low educational level had better compliance [29-31]. It may be presumed that those with higher level of education are better aware of the complications of diabetes and thus are more motivated to adhere to medications and lifestyle modifications. Socioeconomic status may influence diabetes management and control since it is often associated with access to health care, healthcare utilization, use of medication, and access to good nutrition [32]. In Nigeria, only a small proportion of the citizens have prepaid health care through health insurance schemes [33], thus majority pay out of pocket for health care related expenses, thus their affordability of medications is dependent on their socioeconomic status. Diabetic neuropathy was present in almost half of the study population while the prevalence of other diabetic complications were much lower in this study; however, there was no significant association between the number of complications which can be attributed to better diabetes control from self-management of DSMQ scores.

### Limitation

Limitations of this study include its small sample size and that it was a single site study. A larger sample would have improved the power of this study and the findings will be more generalisable. However, the response rate and HOLTER index for this study were within acceptable limits and so the findings reported will be useful in practice, particularly in like populations.

## Conclusion

The Diabetes Self-Management Questionnaire (DSMQ) is a relatively new tool used to assess diabetes self-management. The scale covers several important domains; diet, medication adherence, blood glucose monitoring, physical activity and contact with health-care professionals. The Diabetes Self-Management Questionnaire showed significant correlation with glycaemic control amongst Nigerian diabetics, with individual DSMQ domains of medication adherence, blood glucose monitoring and dietary control showing high correlation with HbA1c. Male sex, and higher educational level are associated with better self-management indices and glycaemic control. Higher income is an independent determinant of good self-management. Results from this study contribute to knowledge, and should inform upstream and downstream public health interventions geared towards improving the control of diabetes mellitus in Nigerian communities.

### What is known about this topic

- Self-management is one of the most important factors contributing to achieving euglycaemia;
- The DSMQ is a reliable and valid instrument that enables an efficient assessment of self-care behaviours associated with glycaemic control.

### What this study adds

- The Diabetes Self-Management Questionnaire showed significant correlation with glycaemic control amongst Nigerian diabetics with individual DSMQ domains of medication adherence, blood glucose monitoring and dietary control showing high correlation with HbA1c;
- Male sex, and higher educational level are associated with better self-management indices and glycaemic control;
- Higher income is an independent determinant of good self-management.
